# Estimated pulse wave velocity as an integrated hemodynamic risk indicator in obese women with gestational hypertension: A cross‐sectional study

**DOI:** 10.14814/phy2.71078

**Published:** 2026-08-20

**Authors:** Longa Kaluba, Ellah Banda Nyambe, Mordecai Malambo, Theresa Chikopela, Moses Mukosha, Kenneth Chanda, Karen Imasiku, Kate Bramham, Fastone Goma

**Affiliations:** ^1^ Physiological Sciences Department, School of Medicine University of Zambia Lusaka Zambia; ^2^ Adult Infectious Disease Hospital University Teaching Hospital Lusaka Zambia; ^3^ Cavendish University Zambia School of Medicine Lusaka Zambia; ^4^ Department of Pharmacy, School of Health Sciences University of Zambia Lusaka Zambia; ^5^ Department of Obstetrics and Gynaecology, School of Medicine University of Zambia Lusaka Zambia; ^6^ Division of Women's Health, Women's Health Academic Centre King's College London and King's Health Partners, St Thomas' Hospital London UK; ^7^ Centre for Primary Care Research Lusaka Zambia

**Keywords:** arterial stiffness, carotid radial pulse wave velocity, gestational hypertension (GH), obesity

## Abstract

Hypertensive disorders of pregnancy are associated with increased cardiovascular risk and arterial remodeling, further complicated by obesity. While carotid‐femoral pulse wave velocity remains the gold standard for arterial stiffness assessment, estimated PWV (ePWV) and carotid‐radial PWV (crPWV) offer practical alternatives. This study evaluated ePWV and crPWV in gestational hypertension (GH) and their association with asymmetric dimethylarginine (ADMA). Cross‐sectional analysis of 74 obese pregnant women (GH: *n* = 38; normotensive: *n* = 36). Lifestyle characteristics were assessed via WHO STEPS questionnaire. Seated blood pressure was measured and ePWV calculated. crPWV and augmentation index (AIx) were assessed using Complior Analyse. Serum ADMA was measured. Multivariable linear regression identified independent predictors of arterial stiffness, adjusting for central systolic blood pressure (cSBP), AIx, ADMA, and GH status. ePWV was significantly higher in the GH group (7.7 vs. 6.6 m/s; *p* < 0.001), while crPWV did not differ significantly (*p* = 0.14). A significant correlation between ePWV and crPWV was observed (*r* = 0.42; *p* < 0.001). ADMA did not differ between groups. cSBP and AIx independently predicted crPWV; GH status independently predicted ePWV. ADMA was not significantly associated with either marker. ePWV may serve as a practical, effective index for assessing vascular load in obese obstetric populations.

## 1 INTRODUCTION

Hypertensive disorders of pregnancy (HDPs)—comprising chronic hypertension, gestational hypertension, and pre‐eclampsia—remain a leading cause of maternal and foetal morbidity and mortality worldwide (Braunthal & Brateanu, 2019). While hypertension represents the most significant modifiable driver of global mortality, nearly 50% of the hypertensive population remains undiagnosed (WHO, 2023). This burden is particularly acute in Low‐ and Middle‐Income Countries (LMICs), which account for 73% of all premature deaths related to non‐communicable diseases (WHO, 2024). Locally, the prevalence of preeclampsia is notably high at 13.8% (Ying et al., 2023). Within this landscape, cardiovascular disease has emerged as the primary cause of death among women globally, necessitating a more nuanced understanding of vascular health in maternal populations. Beyond the immediate peripartum risks, women with a history of HDPs exhibit a significantly higher lifetime risk of cardiovascular disease (CVD), including chronic hypertension, stroke, and ischemic heart disease (Xu et al., 2022).

Central to the pathophysiology of HDPs is vascular dysfunction, characterized by increased arterial stiffness. Pulse wave velocity (PWV) is a well‐established, noninvasive measure of arterial stiffness and an independent predictor of cardiovascular events, with carotid femoral PWV (cfPWV) recognized as the gold standard (Fourie et al., 2020; Vlachopoulos et al., 2010). However, the measurement of carotid‐radial PWV (crPWV)—a measure of muscular artery stiffness—is particularly useful in pregnancy when central measurements are anatomically restricted. Unlike cfPWV, which requires difficult groin access and uncomfortable supine positioning—challenges often exacerbated by adipose tissue dampening the pulse wave—crPWV offers a less invasive alternative. It bypasses the technical barriers from the gravid abdomen and allows for measurements in the left lateral position, significantly enhancing patient comfort and feasibility.

While crPWV provides a direct physical measurement, estimated PWV (ePWV)—calculated from age as a proxy for structural stiffness (elastin loss) and mean arterial pressure for functional stiffness (wall tension)—has gained traction as a cost‐effective, pressure‐ and age‐based surrogate correlate of central aortic stiffness (Greve et al., 2016; Reference Values for Arterial Stiffness Collaboration, 2010). Given the metabolic and haemodynamic risk profile of our study population, we applied the ePWV equation validated for individuals with cardiovascular risk factors rather than the healthy‐population reference model. However, the degree to which these two metrics, crPWV and ePWV, correlate in the acute setting of hypertensive pregnancy remains poorly understood.

Compounding this clinical picture is the global rise in maternal obesity. Indeed, the risk of developing HDPs doubles for overweight pregnant women with a BMI of 26 kg/m^2^ and almost triples for obese pregnant women with a BMI of 30 kg/m^2^ (Lopez‐Jaramillo et al., 2018). Obesity itself is an independent driver of systemic inflammation and oxidative stress, both of which contribute to arterial stiffening (Bohiltea et al., 2020). A potential mediator in this process is asymmetric dimethylarginine (ADMA), an endogenous inhibitor of nitric oxide synthase. By inhibiting endothelial nitric oxide synthase (eNOS) and reducing NO bioavailability, ADMA has been implicated in promoting increased vascular smooth muscle tone and wall tension, which may contribute to functional arterial stiffness as reflected in elevated PWV (Sibal et al., 2010), though this specific pathway has not been confirmed in pregnant populations.

The present study addressed two aims: to compare ePWV and crPWV between obese pregnant women with and without GH, and to identify independent predictors of each metric, including GH status, augmentation index, central systolic blood pressure, and ADMA.

## 2 METHODS

### 2.1 Study design, population, and setting

**FIGURE 1:**
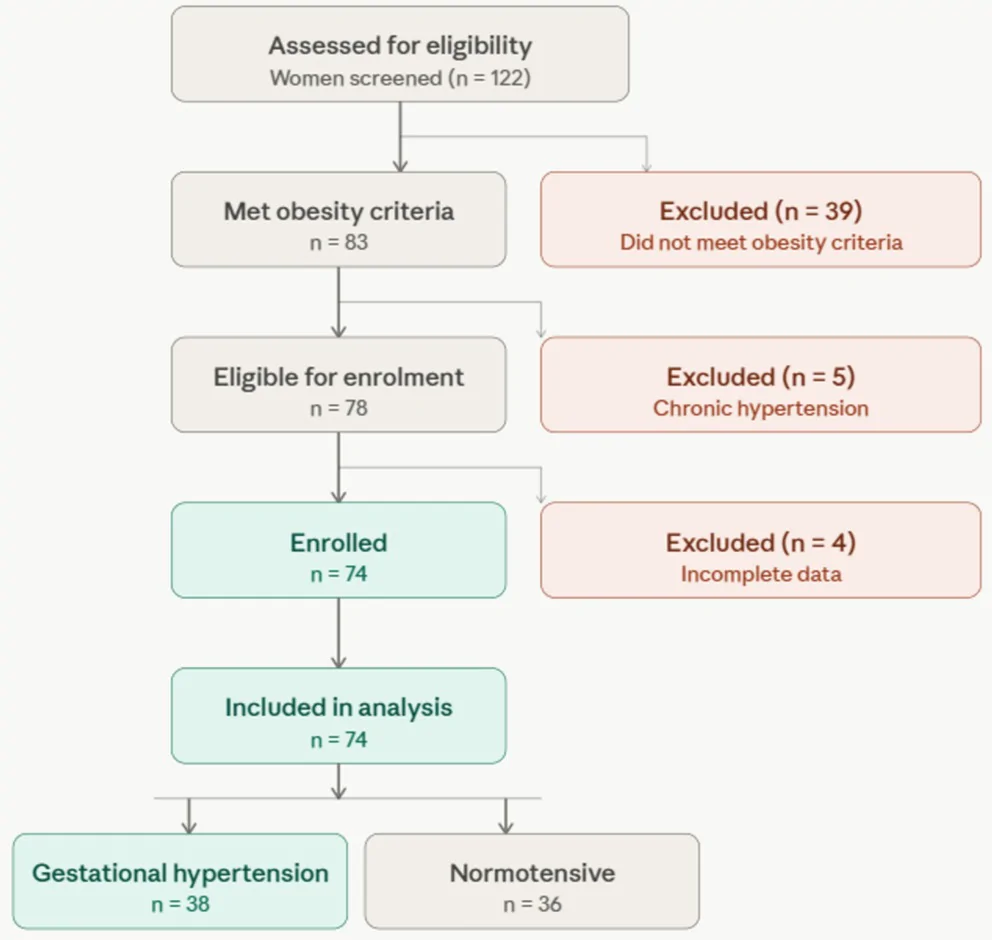
CONSORT flow diagram showing participant screening, exclusion, and enrolment. A total of 122 women were assessed for eligibility. Of these, 39 were excluded for not meeting obesity criteria and five for chronic hypertension, leaving 78 eligible participants. A further four were excluded due to incomplete data, resulting in a final analytic sample of 74 obese pregnant women, allocated to the gestational hypertension (GH; *n* = 38) and normotensive (NT; *n* = 36) groups.

This cross‐sectional study included consecutively recruited obese pregnant women between the ages of 18 and 45 years presenting to the Women and Newborn and Chilenje Level One Hospitals in Lusaka for a routine antenatal clinic visit during the study period (12th December 2024 to 30th July 2025) who met the eligibility criteria and gave consent, either written or verbal, to participate (Figure 1). Verbal consent was witnessed by a member of the research team and documented with a co‐signature on the data collection tool. Eligible participants included obese pregnant women both with and without gestational hypertension, allowing comparison between GH and normotensive (NT) groups. Urine samples were evaluated in all participants as part of the screening and exclusion protocol. Pregnant women were excluded if they had chronic hypertension, diabetes mellitus, known cardiovascular pathology, or pre‐eclampsia, the latter defined as hypertension arising after 20 weeks of gestation with co‐existing proteinuria on dipstick urinalysis (≥1+), consistent with ISSHP guidelines.

### 2.2 Sample size calculation

The assumed 4 m/s crPWV difference was derived from Kaluba et al. (2015), a locally conducted study of crPWV in normotensive and hypertensive pregnant women. While our sample was powered to detect this effect (*β* = 0.80, *α* = 0.05), it was substantially underpowered for the observed group difference of 0.65 m/s (Cohen's *d* = 0.36; post‐hoc power: 31%)—likely reflecting attenuation of the GH‐related crPWV signal in our exclusively obese sample. Consequently, this nonsignificant finding is inconclusive rather than proof of structural equivalence. We cannot rule out a clinically meaningful difference in local arterial stiffness, which limits our ability to determine if the ePWV elevation reflects mathematical coupling alone or a genuine vascular component. Future studies adequately powered for a 0.5–1.0 m/s crPWV difference are required to resolve this.

### 2.3 Data collection

Socio‐demographic data was collected using the WHO STEPS questionnaire (WHO, 2020). crPWV, a direct measure of peripheral arterial stiffness, calculated from the time taken for the arterial pulse to propagate between the carotid and radial arteries, was measured. In addition to crPWV, the augmentation index (AIx) was assessed; AIx represents the enhancement of the central aortic pressure wave by peripheral reflections and serves as a sensitive marker of systemic vascular compliance (Afsar & Elsurer, 2014). All parameters were recorded using the Alam Complior Analyse device (Alam Medical, France). The distance between arterial sites was measured to the nearest millimeter using a clinical tape measure. The carotid‐radial anatomical distance was determined by using a nonelastic tape measure to record the direct, straight‐line distance from the common carotid artery site to the radial artery site at the wrist. Measurements were obtained via noninvasive pressure transducers applied to the skin surface over the carotid and radial arteries, and the software applied the recommended subtraction method (distance * 0.8) to estimate the true aortic path length, in accordance with the ARTERY Society expert consensus recommendations for standardized PWV measurement (Van Bortel et al., 2012). Pulse wave acquisition was accepted only when the device's internal signal quality index confirmed stable, reproducible waveforms over a minimum of 10 consecutive cardiac cycles. Each participant had two measurements taken; to ensure optimal data quality, only the measurement exhibiting the lowest tolerance index was analyzed. To ensure hemodynamic stability and avoid aortocaval compression, participants were positioned in a left lateral recumbent position after a minimum 5‐min rest period. During data acquisition, participants were instructed to remain still and refrain from speaking or sleeping.

Venous blood samples were collected from all participants to assess serum concentrations of ADMA. Samples were centrifuged for 10 min at 1000 × g, after which serum was aliquoted and stored at −80°C to ensure analyte stability until the time of analysis. Maternal ADMA concentrations were quantified using a high‐sensitivity Enzyme‐Linked Immunosorbent Assay (ELISA) kit (Elabscience, China, Catalogue number: E‐EL‐0042; Sensitivity: 9.38 ng/mL), following the manufacturer's protocol. The assay demonstrated high precision, with manufacturer‐reported intra‐assay and inter‐assay coefficients of variation (CV) of <10%.

#### 2.3.1 Definitions

1. Gestational hypertension (GH): any occurrence of either systolic blood pressure (SBP) ≥140 mmHg or diastolic blood pressure (DBP) ≥90 mmHg starting at or after 20 weeks of gestation among women with previously normal blood pressure before 20 weeks.
2. Obesity: pre‐pregnancy body mass index of 30 Kg/m^2^ or greater.
3. ePWV = 9.587 − (0.402 × age) + (4.560 × 10^−3^ × age^2^) − (2.621 × 10^−5^ × age^2^ × MBP) + (3.176 × 10^−3^ × age × MBP) − (1.832 × 10^−2^ × MBP), ePWV was calculated using the polynomial equation derived from the reference value population (individuals with cardiovascular risk factors) as described by the reference values for arterial stiffness collaboration (Greve et al., 2016; Reference Values for Arterial Stiffness Collaboration, 2010). This specific equation was chosen over the “healthy population” model to more accurately reflect the metabolic and hemodynamic risk profile of our overweight and hypertensive population.

### 2.4 Statistical analysis

Data analysis was performed using Stata Version 15.1 (StataCorp, College Station, TX, USA). Five participants with missing crPWV values were excluded from crPWV analyses only. Missingness was not significantly associated with group allocation (*p* = 0.071), supporting a missing‐at‐random assumption. No formal sensitivity analysis was therefore performed. Continuous variables were assessed for normality using the Shapiro–Wilk test and visual inspection of histograms. Descriptive statistics are presented as mean ± standard deviation (SD) for normally distributed data, or median (interquartile range) for nonparametric data.

Clinical and demographic characteristics between the NT and GH groups were evaluated using independent *t*‐tests or Mann–Whitney *U* tests, as appropriate. Categorical variables were compared using the chi‐square test. The relationship between arterial stiffness metrics (ePWV and crPWV) and clinical/biochemical markers was first assessed using Pearson's correlation coefficients.

Multivariable linear regression models were constructed to examine the relationship between GH status and arterial stiffness, with ePWV (Model 1) and crPWV (Model 2) as outcomes. The primary models adjusted for AIx and ADMA only, deliberately excluding blood pressure variables. An exploratory analysis was also conducted, adjusted for cSBP, to examine the independent contribution of blood pressure. Multicollinearity was assessed using the variance inflation factor (VIF), with a threshold of <10 considered acceptable. A two‐tailed *p* value <0.05 was considered statistically significant for all analyses.

### 2.5 Ethical considerations

Participants provided written informed consent, and approval was obtained from the University of Zambia Biomedical Research Ethics Committee (UNZABREC reference number 5604‐2024) and the National Health Research Authority (NHRA‐1471/15/08/2024/13/08/2024). Human participant involvement complied fully with institutional standards and the ethical principles outlined in the Declaration of Helsinki. Venous blood was collected via venipuncture by trained clinical personnel in accordance with institutional safety protocols. To ensure participant comfort and safety, blood volumes were minimized, and procedures were conducted in a private setting. Samples were immediately pseudonymized and handled according to established biosafety guidelines, with participants retaining the right to withdraw from the procedure without impact on their clinical care.

## 3 RESULTS

A total of 74 obese pregnant women (36 NT and 38 with GH) were recruited from both Women and Newborn and Chilenje Level One Hospitals in Lusaka for a period of 4 months in each institution. Maternal and gestational age and body mass index (BMI) were comparable between groups (Table 1). Both peripheral and central blood pressures, including SBP, DBP, and mean arterial pressure (MAP), were significantly higher in the GH participants. Lifestyle characteristics were comparable between NT and GH groups. Tobacco and alcohol use were negligible (<5% and 1%, respectively) (Table 1). Furthermore, there were no significant differences in dietary patterns or sedentary behavior, with high sedentary risk (≥8 h/day) observed in only 20% of the total participants.

**TABLE 1:** Clinical and demographic participant characteristics.

	NT (36)	GH (38)	*p* Value
Maternal age (years)	31.6 ± 4.5	32.4 ± 5.8	0.49
Gestational age (weeks)	28.0 ± 8.2	30.3 ± 6.2	0.19
BMI (kg/m^2^)	36.5 ± 4.8	36.1 ± 5.1	0.76
Brachial SBP (bSBP) (mmHg)	120.3 ± 12.1	143.0 ± 12.7	**<0.001**
Brachial DBP (bDBP) (mmHg)	76.9 ± 9.3	93.7 ± 10.7	**<0.001**
Brachial PP (bPP) (mmHg)	44 ± 9.5	48.0 ± 11.5	0.11
Brachial MAP (bMAP) (mmHg)	91.2 ± 9.0	109.3 ± 10.8	**<0.001**
Heart Rate (bpm)	80.8 ± 13.9	82.9 ± 18.4	0.61
Central SBP (cSBP) (mmHg)	113.5 ± 11.7	132.9 ± 16.2	**<0.001**
Central DBP (cDBP) (mmHg)	78.1 ± 8.6	93.5 ± 10.8	**<0.001**
Central PP (cPP) (mmHg)	35.4 ± 9.6	39.5 ± 12.7	0.15
Central MAP (cMAP) (mmHg)	92.3 ± 8.7	109.4 ± 10.9	**<0.001**
Current smoker	3 (8.33%)	1 (2.56%)	0.27
Past smoker	0	3 (7.69%)	0.10
Tobacco exposure at home	1 (2.78%)	0	0.30
Tobacco exposure at work	1 (3.03%)	2 (5.56%)	0.76
Current drinker (past 30 days)	1 (2.78%)	1 (2.63%)	0.75
Low fruit/veg intake (<5 servings/day)	3 (8.33%)	8 (20.51%)	0.14
Sitting time (min/day)	300 (120,450)	300 (120,480)	0.71
Sedentary risk, (%)			0.64
Low/Moderate (<8 h)	28 (77.78%)	32 (82.05%)	
High risk (≥8 h)	8 (22.22%)	7 (17.95%)	

*Note*: Values are mean ± standard deviation (SD), median (interquartile range); *n* (percentage); All continuous variables were normally distributed (Shapiro–Wilk *p* > 0.05). Values presented as mean (±SD) compared using the *t*‐test. Categorical variables compared using Chi‐square test. *p* < 0.05 was significant, marked in bold.

Abbreviations: BMI, body mass index; DBP, diastolic blood pressure; GH, gestational hypertensive group; MAP, mean arterial pressure; PP, pulse pressure; SBP, systolic blood pressure.

**FIGURE 2:**
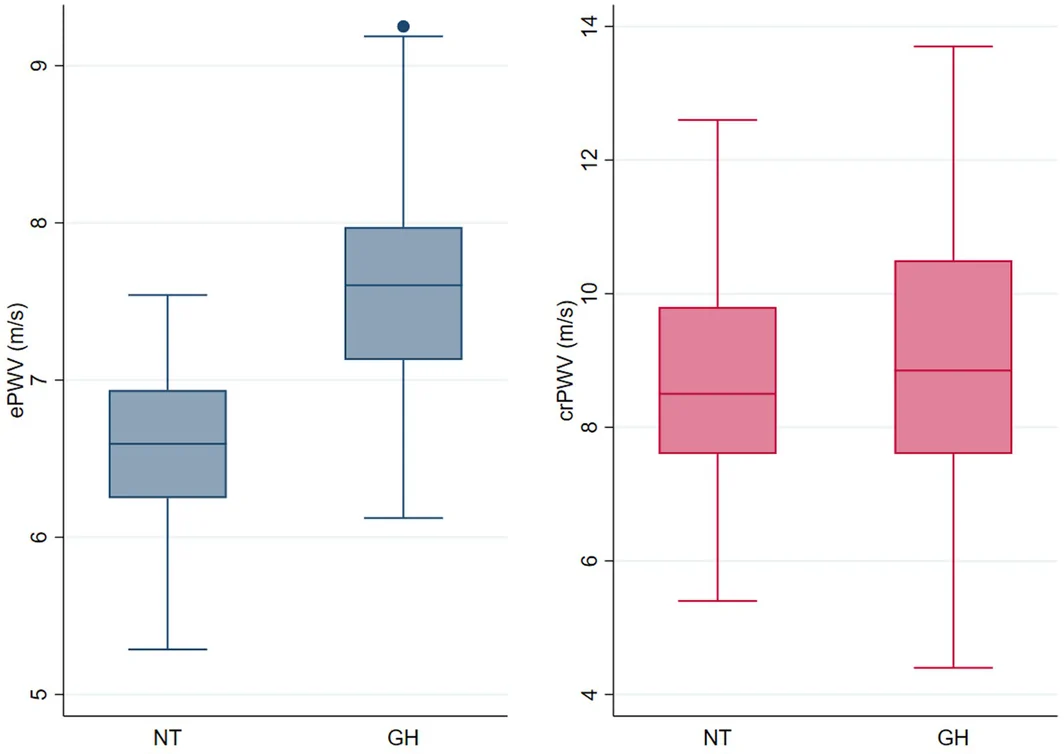
Distribution of estimated pulse wave velocity (ePWV) and carotid‐radial pulse wave velocity (crPWV) by group. Box plots display median and interquartile range; individual data points are overlaid.

The GH group demonstrated significantly higher ePWV (7.7 m/s vs. 6.6 m/s, *p* = <0.001) compared to the NT controls (Figure 2).

However, no significant difference was observed in crPWV (*p* = 0.14), its Alx (*p* = 0.35) and plasma ADMA (0.77 vs. 0.68 μmol/L, *p* = 0.66) compared to NT controls (Table 2). While ePWV was significantly higher in the GH group, this was not explained by differences in smoking, alcohol, or sedentary behavior, as these parameters were also similar across both obese groups.

**TABLE 2:** Vascular markers in NT and GH groups.

	NT (36)	GH (38)	*p* Value
crPWV (m/s)	8.5 ± 1.5	9.2 ± 2.0	0.14
ePWV (m/s)	6.6 ± 0.5	7.7 ± 0.7	**<0.001**
AP (mmHg)	5.9 ± 5.4	7.3 ± 8.6	0.84
Aix (%)	−7.6 ± 19.8	−2.9 ± 21.6	0.35
ADMA	0.68 ± 0.67	0.77 ± 0.79	0.66

*Note*: Most continuous variables were normally distributed (Shapiro–Wilk *p* > 0.05) and compared using the *t*‐test, except AP and ADMA (Mann–Whitney *U* test was used). Values presented as mean (±SD). *p* < 0.05 was significant, marked in bold.

Abbreviations: ADMA, asymmetric dimethylarginine; Alx, augmentation index; AP, augmentation pressure; crPWV, carotid–radial pulse wave velocity; ePWV, estimated pulse wave velocity; GH, gestational hypertensive group.

**FIGURE 3:**
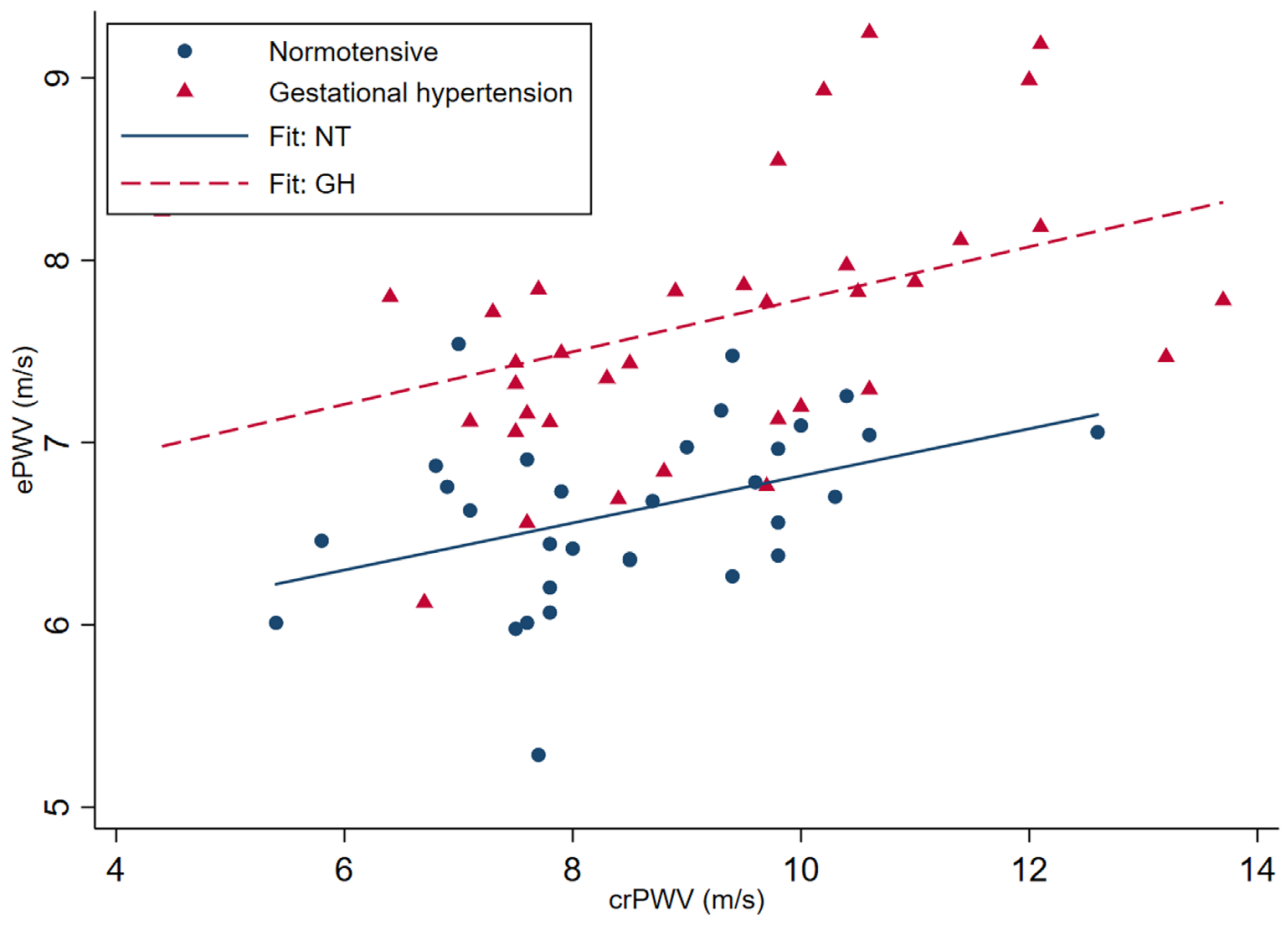
Scatter plot of estimated pulse wave velocity (ePWV) against carotid‐radial pulse wave velocity (crPWV) stratified by group, with group‐specific linear regression lines. A moderate positive correlation between ePWV and crPWV was observed in both normotensive controls (NT: *ρ* = 0.42, *p* = 0.02) and women with gestational hypertension (GH: *ρ* = 0.46, *p* = 0.01), with an overall Spearman correlation of *ρ* = 0.40 (*p* = 0.001). Despite this association, the groups differed significantly on ePWV but not crPWV.

Initial pairwise correlation analysis revealed a weak, nonsignificant positive correlation between ADMA and ePWV (*r* = 0.11, *p* = 0.36) (Table S1). However, a significant moderate correlation was observed between the calculated ePWV and the measured crPWV (*r* = 0.42, *p* = 0.001), suggesting a partial association between central estimated stiffness and peripheral measured stiffness (Figure 3).

**FIGURE 4:**
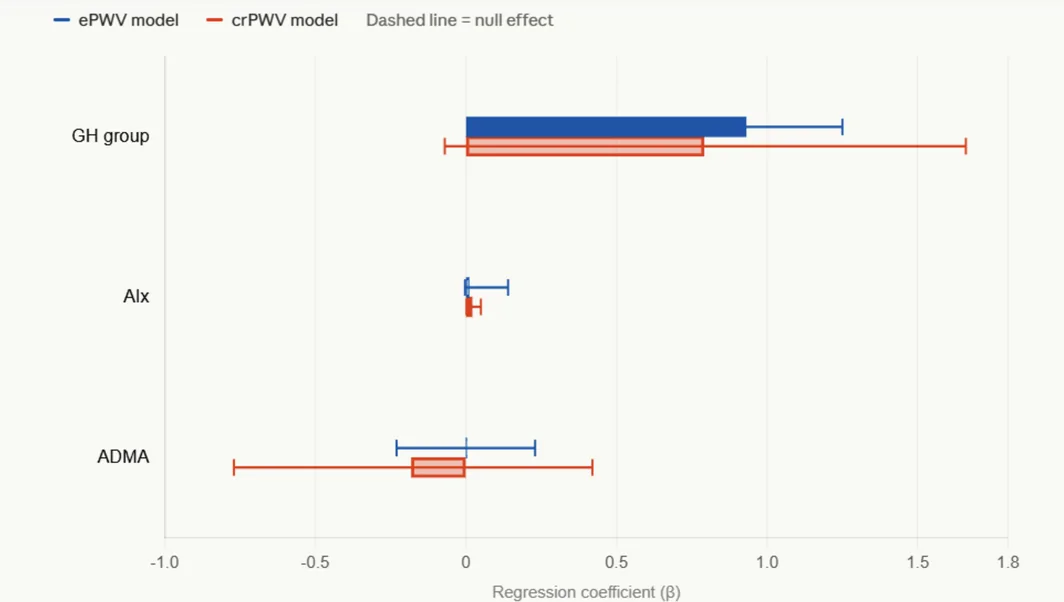
Forest plot of multivariable regression coefficients (*β*) with 95% confidence intervals for predictors of ePWV (blue) and crPWV (orange). Filled markers indicate *p* < 0.05; open markers indicate *p* ≥ 0.05. Horizontal bars represent 95% CIs. The dashed vertical line represents the null effect (*β* = 0).

In the primary model, only GH status was found to be a significant predictor of ePWV (*β* = 0.93, *p* = <0.001). This multivariable model accounted for 41% of the variance in ePWV (*R* ^2^ = 0.41, Prob > *F* = <0.001). In the secondary model (crPWV model), an *R* ^2^ = 0.14 was noted with only Alx emerging as a significant independent predictor (Table 3, Figure 4). Although ADMA was non‐normally distributed, regression model residuals were approximately normally distributed (Shapiro–Wilk *p* = 0.41), supporting the use of standard linear regression without variable transformation.

**TABLE 3:** Multivariable linear regression for factors associated with arterial stiffness.

Predictor variable	ePWV model (*β* [*p* value])	95% CI	crPWV model (*β* [*p* value])	95% CI
GH group	0.93 (**<0.001**)	0.61, 1.25	0.79 (0.07)	−0.07, 1.66
Alx	0.01 (0.17)	−0.003, 0.14	0.02 (**0.03**)	0.002, 0.05
ADMA	0.001 (0.99)	−0.23, 0.23	−0.18 (0.56)	−0.77, 0.42
Model *R* ^2^	0.41		0.14	
Model Prob > *F*	**<0.001**		**0.03**	

*Note*: *p* < 0.05 was significant, marked in bold.

Abbreviations: ADMA, Asymmetric Dimethylarginine; AIx, Augmentation index; crPWV, carotid–radial pulse wave velocity; ePWV, estimated pulse wave velocity.

### 3.1 Explorative analysis

After adjusting for Alx, cSBP and GH status, ADMA was not found to be a significant predictor of ePWV (*β* = −0.01, *p* = 0.9). This multivariable model accounted for 75% of the variance in ePWV (*R* ^2^ = 0.75, Prob > *F* = <0.001) (Table 4). Furthermore, post‐estimation diagnostics confirmed the absence of multicollinearity (Max VIF = 1.23). In the secondary model, (crPWV model), an *R* ^2^ = 0.22 was noted with GH status not emerging as a significant independent predictor after adjusting for Alx, cSBP, and ADMA. However, similar to the ePWV findings, both cSBP (*β* = 0.04, *p* = 0.02) and Alx (*β* = 0.03, *p* = 0.01) were identified as significant predictors of arterial stiffness (Table 4).

**TABLE 4:** Multivariable linear regression for factors associated with arterial stiffness, including blood pressure.

Predictor variable	ePWV model (*β* [*p* value])	95% CI	crPWV model (*β* [*p* value])	95% CI
GH group	0.34 (**0.01**)	0.10, 0.59	0.12 (0.81)	−0.87, 1.11
Alx	0.01 (**0.01**)	0.002, 0.01	0.03 (**0.01**)	0.01,0.05
cSBP	0.03 (**<0.001**)	0.03, 0.04	0.04 (**0.02**)	0.01,0.07
ADMA	−0.01 (0.94)	−0.15, 0.14	−0.19 (0.50)	−0.76, 0.38
Model R^2^	0.75		0.22	
Model Prob > F	**<0.001**		**0.01**	

*Note*: *p* < 0.05 was significant, marked in bold.

Abbreviations: ADMA, Asymmetric Dimethylarginine; AIx, Augmentation index; crPWV, carotid–radial pulse wave velocity; ePWV, estimated pulse wave velocity.

## 4 DISCUSSION

Our findings suggest ePWV, derived from age and blood pressure alone, represents a feasible and zero‐cost indicator of elevated vascular load in obese women with GH, and may complement routine blood pressure monitoring in antenatal settings. While crPWV reflects the stiffness of the peripheral, muscular arteries, ePWV is intended to approximate pressure‐related load in the central, elastic arteries. The significant association between ePWV and GH status suggests that the systemic impact of GH in this population manifests primarily as increased pressure‐related vascular load, rather than arterial stiffening in the muscular arteries captured by crPWV.

Although the 1.1 m/s difference in ePWV between groups was statistically significant, its clinical meaningfulness warrants careful consideration. Pregnancy‐specific ePWV reference ranges have not been formally established, limiting threshold‐based interpretation. The normotensive mean of 6.6 m/s falls within values reported for healthy pregnant women, while the GH mean of 7.7 m/s approaches but does not clearly exceed the 9.0 m/s threshold associated with adverse pregnancy outcomes in comparable cohorts (An et al., 2023).

The exploratory model incorporating systolic blood pressure (*R* ^2^ = 0.75) should be interpreted with caution. Because ePWV is mathematically derived from blood pressure and age, and GH is itself defined by elevated blood pressure, a substantial proportion of the explained variance in this model reflects mathematical circularity rather than independent physiological prediction.

AIx reflects the intensity of wave reflections originating in the microvasculature not fully reflected in stiffness metrics alone (Anthoulakis & Mamopoulos, 2022). In patients with GH, particularly those with obesity, factors such as chronic low‐grade inflammation and sympathetic overactivity drive an increase in peripheral vascular tone. Notably, AIx was a significant predictor of crPWV but not ePWV in the primary model, suggesting that directly measured vascular indices are more sensitive to the haemodynamic effects of wave reflections within the arterial tree. While ePWV provides a pressure‐derived estimate of aortic stiffness, crPWV reflects the structural mechanical properties of the muscular conduit arteries, and is less influenced by acute haemodynamic state. An elevation in crPWV in GH would therefore suggest structural arterial remodeling that is not solely attributable to the hypertensive pressure load itself. This distinction is particularly relevant in our study population, where obesity‐related vascular changes may independently contribute to arterial wall remodeling in the context of HDP. Indeed, research suggests that the likelihood of developing pre‐eclampsia increases by 89% with each unit increase in BMI (Ying et al., 2023). Our data is consistent with the hypothesis that while obesity elevates clinical risk, its associated vascular effects may be more functional than structural, manifesting as increased tone rather than permanent vessel wall damage.

The lack of a significant difference in crPWV between women with GH and NT controls must be interpreted cautiously, given the 31% achieved power for this comparison. However, null results have been reported in other studies (Phan et al., 2023; Xu et al., 2024). The fact that crPWV measures the carotid‐radial (muscular) path directly suggests that while women with GH exhibit higher functional risk (as reflected by ePWV), they may not yet have developed the structural vascular remodeling or permanent stiffening required to alter physical crPWV measurements. Whether structural changes are absent or confined to central elastic arteries remains to be established in longitudinal studies.

Our findings are consistent with evidence from large obesity cohorts, where ePWV has demonstrated good agreement with MRI‐assessed PWV (van Hout et al., 2022) and has independently predicted all‐cause and cardiovascular mortality, with an AUC exceeding 0.80 in individuals with obesity (Li et al., 2023). Together, these findings support the clinical utility of ePWV as a risk stratification tool in populations where direct PWV measurement is logistically constrained. However, our findings contrast with a previous study that reported significant differences in crPWV within a non‐obese African GH population (Kaluba et al., 2015). We speculate that the discrepancy between the two studies may reflect pre‐existing vascular dysfunction in obesity—driven by adipokine dysregulation and chronic inflammation—which could mask any additional GH‐related structural stiffening that might otherwise be detectable in leaner women with healthier vasculature. This would be consistent with our finding that functional (ePWV) differences were present, while structural (crPWV) differences were not.

Vascular health is largely dependent on the homeostatic role of nitric oxide (NO). Systemic inflammation and metabolic disease disrupt this balance by increasing ADMA levels, a potent inhibitor of NO production. By limiting NO availability, ADMA promotes endothelial impairment. Its clinical significance is well‐documented across multiple pathologies where it serves as a primary predictor of long‐term cardiovascular outcomes and mortality, independent of conventional risk factors (Sibal et al., 2010). Previous research indicates that ADMA levels are elevated as early as the first trimester in patients predisposed to pre‐eclampsia, highlighting its role as a precursor to clinical symptom onset (Bian et al., 2015). Further, preliminary evidence identifies ADMA as a potential indicator of disease trajectory in the early stages of chronic kidney disease (Suryantoro et al., 2025). Despite the known association between ADMA and endothelial dysfunction, ADMA did not emerge as a significant predictor of ePWV or crPWV in either of our regression models. This null finding should be interpreted cautiously given the cross‐sectional design, modest sample size, and the likelihood that the study was underpowered to detect a subtle ADMA‐stiffness association. Whether ADMA contributes to vascular stiffening through pathways not captured by these metrics or whether its role is confined to earlier or later stages of vascular compromise remains an open question requiring longitudinal investigation with larger samples and central arterial stiffness measurement.

### 4.1 Strengths and limitations

By focusing exclusively on obese women, this study minimizes the confounding effect of varying adiposity levels, allowing for a more specific exploration of how GH and ADMA interact within this high‐risk metabolic environment. The use of both ePWV and measured crPWV stiffness provides a rare opportunity to distinguish between formula‐driven cardiovascular risk and actual physical vascular remodeling. Lastly, the use of multivariable regression and VIF testing ensures that the reported associations (and lack thereof) are robust and not merely the result of multicollinearity.

The primary limitation of this study is its cross‐sectional design, which precludes causal inference or conclusions about temporal sequence across all reported associations. Specifically, it cannot be determined whether elevated ePWV precedes structural arterial stiffening or whether the null crPWV finding reflects true structural equivalence or insufficient follow‐up time for remodeling to manifest.

Further, crPWV comparison was inadequately powered. The study was originally powered to detect a 4 m/s difference in crPWV, substantially exceeding the observed difference of 0.65 m/s (Cohen's *d* = 0.36). Post hoc power analysis confirmed only 31% achieved power, indicating the non‐significant crPWV finding reflects insufficient statistical resolution rather than true equivalence. Furthermore, the absence of a nonobese control group was another limitation. Because our entire population was obese, systemic inflammation and oxidative stress were likely elevated at baseline across all participants, potentially producing a “saturation” effect that may have masked ADMA's predictive power, an effect that might only become apparent when lean and obese subjects are compared directly. Without that comparison, we cannot determine whether the null ADMA finding reflects obesity‐driven vascular saturation, a genuine absence of ADMA‐stiffness association in pregnancy, or both. Also, the study was conducted across two urban hospitals in Lusaka, limiting generalisability to rural Zambian populations and other African settings where vascular risk profiles, healthcare access, nutritional status, and obstetric care pathways may differ substantially. While crPWV provides a direct device‐measured index of peripheral muscular artery stiffness, it does not assess the central elastic aortic pathway. The absence of carotid‐femoral PWV—the gold‐standard measure of central arterial stiffness and the reference against which ePWV was originally validated—represents a significant limitation, precluding formal assessment of ePWV's surrogate validity in this specific population.

## 5 CONCLUSION

Our findings suggest that ePWV, calculated from routinely collected age and blood pressure data, is a feasible and zero‐cost indicator of elevated vascular load in obese women with GH. Prospective studies are needed to validate its performance as a clinical screening tool in antenatal care settings. While ePWV reflects an integrated vascular risk index driven by pressure load and age, crPWV captures peripheral structural vascular remodeling; the two metrics are complementary, each providing a distinct window into vascular health in GH.

## AUTHOR CONTRIBUTIONS

**Longa Kaluba:** Conceptualization; data curation; formal analysis; funding acquisition; investigation; methodology; project administration. **Ellah Banda Nyambe:** Methodology; project administration; resources; visualization. **Mordecai Malambo:** Data curation; formal analysis; methodology. **Theresa Chikopela:** Formal analysis; software; validation. **Moses Mukosha:** Formal analysis; methodology; validation. **Kenneth Chanda:** Investigation; project administration. **Karen Imasiku:** Investigation; methodology. **Kate Bramham:** Investigation; validation. **Fastone Goma:** Conceptualization; supervision; validation.

## FUNDING INFORMATION

This work was supported by the Norwegian Programme for Capacity Development in Higher Education and Research for Development (NORHED II) project (60260).

## CONFLICT OF INTEREST STATEMENT

The authors declare that they have no financial or personal relationships that may have inappropriately influenced them in writing this article.

## Supporting information


Table S1.

## Data Availability

The data that support the findings of this study are available from the corresponding author, L.K., upon reasonable request.
