# Supplementary material for: Estimated pulse wave velocity as an integrated hemodynamic risk indicator in obese women with gestational hypertension: A cross‐sectional study
Source: Physiol Rep. 2026 Aug 20;14(16):e71078. doi: 10.14814/phy2.71078 (PMC13494149; doi:10.14814/phy2.71078)
Supplement: Supplementary file 1 [file PHY2-14-e71078-s001.docx]

Supplementary Table S1. Pearson correlation matrix of all continuous variables

|  | **ePWV** | **crPWV** | **AIx** | **ADMA** | **Age** | **BMI** | **cMAP** | **cSBP** | **cDBP** |
| --- | --- | --- | --- | --- | --- | --- | --- | --- | --- |
| **ePWV** |  |  |  |  |  |  |  |  |  |
| **crPWV** | **0.42**** | — |  |  |  |  |  |  |  |
| **AIx** | **0.26*** | **0.27*** | — |  |  |  |  |  |  |
| **ADMA** | 0.11 | -0.08 | 0.14 | — |  |  |  |  |  |
| **Age** | **0.29*** | 0.23 | 0.13 | 0.20 | — |  |  |  |  |
| **BMI** | −0.05 | −0.08 | −0.16 | −0.01 | 0.04 | — |  |  |  |
| **cMAP** | **0.95**** | **0.43**** | 0.23 | 0.05 | 0.01 | 0.01 | — |  |  |
| **cSBP** | **0.84**** | **0.32**** | 0.05 | -0.01 | 0.10 | 0.05 | **0.85**** | — |  |
| **cDBP** | **0.91**** | **0.44**** | 0.23 | 0.01 | -0.03 | −0.04 | **0.96**** | **0.74**** | — |

*p<0.05; **p<0.01. Values are Pearson rho coefficients except for ADMA (Spearman was used). Significant correlations in bold

Supplementary Table S1. Pearson correlation matrix of continuous study variables (N=75). Upper triangle omitted by symmetry. ePWV = estimated pulse wave velocity; crPWV = carotid-radial pulse wave velocity; AIx = augmentation index; ADMA = asymmetric dimethylarginine; cMAP = central mean arterial pressure; cSBP = central systolic blood pressure; cDBP = central diastolic blood pressure. *p<0.05; **p<0.01.
